# A40 GUT MICROBIOTA PROMOTES NUTRIENT AVAILABLITY AND PATHOGENESIS OF AN ATTACHING/ EFFACING BACTERIAL PATHOGEN

**DOI:** 10.1093/jcag/gwac036.040

**Published:** 2023-03-07

**Authors:** Q Liang, J Allaire, L Celiberto, H Yu, B Vallance

**Affiliations:** 1 Experimental Medicine, University of British Columbia; 2 Pediatrics, BC Children's Hospital Research Institute, Vancouver, BC; 3 The Janssen Pharmaceutical Companies of Johnson & Johnson, Montreal, QC, Canada

## Abstract

**Background:**

Our gut microbiota plays an important role in protecting the gastrointestinal (GI) tract from invading enteric pathogens. Much of this colonization resistance is mediated by limiting nutrient availability, however, enteric pathogens have evolved strategies to subvert this competition, utilizing commensal metabolites to facilitate their infection. Access to nutrients is not only crucial for a pathogen’s metabolic fitness, but can also drive the expression of virulence factors, a process high in energy demands. In addition, enteric pathogens, such as the attaching and effacing (A/E) bacterium *Citrobacter rodentium*, must cross the colonic mucus layer that normally prevents their direct access to the underlying epithelium. Intestinal mucus is comprised of highly glycosylated mucins, with the sugar sialic acid frequently occupying the terminal position of their *O*-glycan side chains. We hypothesize that *C. rodentium* utilizes commensal-liberated mucin sugars, such as sialic acid, as nutrients and signals to promote its virulence.

**Purpose:**

This study investigates the mechanisms by which A/E pathogens reach the colonic mucosal surface, and the role played by commensal microbes in facilitating the infection.

**Method:**

Expression of virulence factors secreted by *C. rodentium* in the presence or absence of sialic acid was analyzed by SDS-PAGE and mass spectrometry. Next, we infected specific-pathogen free (SPF), germfree (GF), and previously GF C57Bl/6 mice mono-colonized with *Bacteroides thetaotaomicron*, a mucus-degrading commensal, to examine their susceptibility to *C. rodentium* and to measure the levels of free sialic acid in their feces.

**Result(s):**

Sensing of sialic acid by *C. rodentium*, was found to induce the secretion of several key virulence proteins, enhancing the pathogen’s migration across the colonic mucus layer and adhesion to the underlying epithelium. Access to sialic acid within the gut environment was enhanced in the presence of microbiota, as the levels of free sialic acid were low in GF mice. Interestingly, despite GF mice carrying very high *C. rodentium* burdens, passage across the mucus layer and infection of their colonic epithelium was impaired as compared to SPF mice. Notably, *B. thetaotaomicron* was found to degrade whole mucus in vitro, facilitating its consumption by *C. rodentium* for growth, while *B. thetaotaomicron* mono-colonized GF mice showed increased susceptibility to colonic infection by *C. rodentium*.

**Conclusion(s):**

We demonstrate that although commensal microbes promote colonization resistance, as an A/E pathogen infection establishes, specific commensal bacteria accelerate infection in the GI tract by releasing an important nutrient, ie. sialic acid, from mucus. Access to sialic acid promotes *C. rodentium* virulence by inducing the key virulence factors that facilitate its translocation across the mucus layer as well as adhesion to the epithelium, thereby expediting disease progression.

**Please acknowledge all funding agencies by checking the applicable boxes below:**

CCC, CIHR, Other

**Please indicate your source of funding;:**

CH.I.L.D. Foundation

**Disclosure of Interest:**

None Declared

